# Central Line Catheters and Associated Complications: A Review

**DOI:** 10.7759/cureus.4717

**Published:** 2019-05-22

**Authors:** Avani R Patel, Amar R Patel, Shivank Singh, Shantanu Singh, Imran Khawaja

**Affiliations:** 1 Internal Medicine, Northern California Kaiser Permanente, Fremont, USA; 2 Internal Medicine, Southern Medical University, Guangzhou, CHN; 3 Pulmonary Medicine, Marshall University School of Medicine, Huntington, USA

**Keywords:** central venous catheterization, subclavian vein cannula insertion, subclavian vein cannula insertion, pneumothorax, infection, bleeding, complication, complications, catheter knot

## Abstract

The use of a central line or central venous catheterization was brought to attention in 1929 when Dr. Werner Forssmann self-inserted a ureteric catheter through his cubital vein and into the right side of his heart. Since that time the central line technique has developed further and has become essential for the treatment of decompensating patients. Central lines are widely used for anything from rapid fluid resuscitation, to drug administration, to parenteral nutrition, and even for administering hemodialysis. Central lines come in different sizes, types, and sites of administration. Sometimes their use can be associated with complications as well. The following review article addresses these parameters of central lines and goes into detail regarding their complications.

## Introduction and background

**Figure 1:**
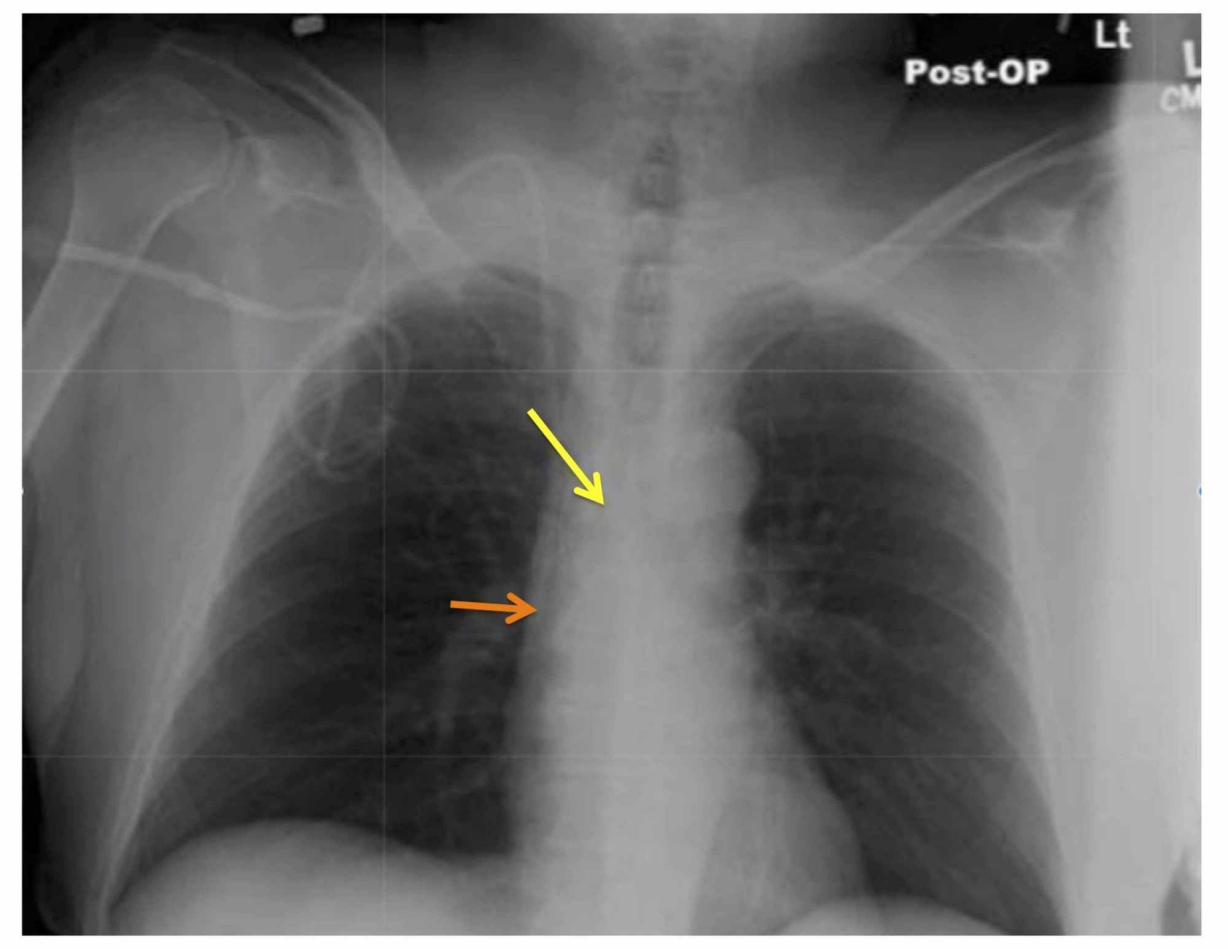
Chest radiograph showing a central line This is a right-sided central line in internal jugular vein (the tip is pointed out by the orange arrow). The central line is positioned near the right tracheobronchial angle (pointed out by the yellow arrow) [3].

The use of a central line is often crucial to the improvement of critically ill patients. The reasons a physician might decide that a patient needs a central line would be to deliver multiple medications, for continuous infusion chemotherapy, for parenteral nutrition, and to deliver vesicant drugs (Figure 1) [1]. Vesicant drugs are medications that can cause serious damage to the skin and muscle tissue if the drug comes into contact with them. Due to this, it would be safer to deliver the vesicant drugs through a central line rather than through a peripheral line where there is a higher chance of drug leakage. The three common sites are the subclavian vein, the internal jugular vein, and the femoral vein. The ideal catheterization site would be one that has less thrombosis, lower infection rates, and fewer mechanical complications [2]. Because of this, the femoral vein is avoided because of a higher rate of infection and thrombosis as compared to the subclavian vein [2]. The subclavian vein tends to have a lower infection rate as compared to other central line sites [2]. This is a review article that describes central lines, their different types, the sites of insertion, and the complications seen in association with them.

## Review

Types of catheters

The choice and selection of the catheter among the available options depend largely on the nature and duration of intended treatment.

Non-tunneled Catheters

Catheters that are non-tunneled are seen and used most frequently in emergency and acute situations. This is because of their simplicity and easy insertion [2]. The concern that physicians have with non-tunneled catheters is that they have a short duration of use. Because of this, they should be removed as soon as possible in order to prevent complications like infections or thrombosis [2].

Tunneled Catheters

Tunneled catheters are preferred when intravenous access is needed for multiple times during a time period longer than one month [2]. They have a lower infection rate as compared to non-tunneled catheters [2].

Implanted Ports

For long-term use, implanted ports are preferred as they have better cosmetic results and less infection as compared to non-tunneled catheters and tunneled catheters. The concern with implanted ports is that they require surgical implantation that takes more time and skill to implant than the other catheter types [2].

Dialysis Catheters

Dialysis catheters are utilized for hemodialysis and continuous renal replacement techniques (CRRT) [4]. Both are used for filtration of the blood, usually in medical emergencies like acute kidney injury, severe sepsis, fluid overload, or septic shock [5]. The other indications for dialysis include metabolic acidosis, hyperkalemia, the ingestion of certain drugs (salicylates, lithium, isopropanol, methanol, and ethylene glycol), fluid overload, uremia, and when the serum creatinine is greater than 10 mg/dL. The difference between hemodialysis and CRRT is that CRRT will provide a slower fluid removal as compared to intermittent hemodialysis which will lead to better hemodynamic stability in fluid overload patients [4]. 

The central lines for hemodialysis have large bores that require heparin to prevent clotting and may be tunneled for long-term use [2]. For emergent situations, the central line catheters may be non-tunneled for the purpose of easy insertion.

Peripherally Inserted Central Catheters

**Figure 2:**
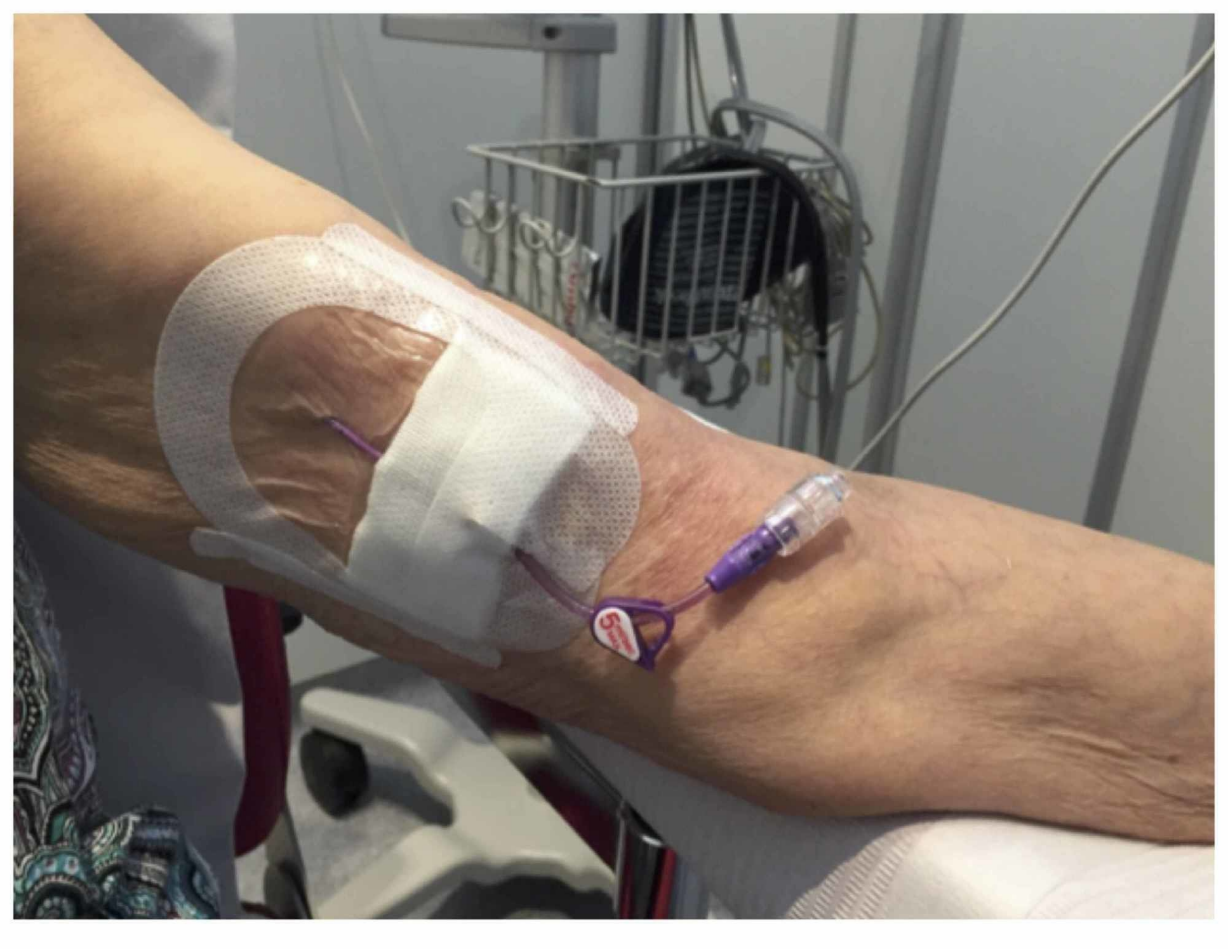
A peripherally inserted central catheter The above figure is a photograph of a patient's cubital fossa showing the insertion site of a peripherally inserted central catheter (PICC) [6].

A peripherally inserted central catheter or a PICC line is a thin, flexible tube that is inserted into an upper arm vein and then guided into the superior vena cava on the right side of the heart. PICC lines can remain inserted for weeks to months (Figure 2) [2]. They are indicated in situations where the patient needs an intravenous delivery of antibiotics or chemotherapy drugs while preserving the integrity of the peripheral vascular system [5]. 

Sites of a central line placement

There are three common sites of central line insertion. These sites are the subclavian vein, the internal jugular vein, and the femoral vein. In most scenarios, establishing central venous access with ultrasound guidance is considered the standard of care [2].

The Subclavian Vein

The subclavian vein is one of the most common sites for central line placement. The subclavian vein is a continuation of the axillary vein (which is a continuation of the brachial vein) [7]. At the lateral border of the first rib, the axillary vein will become the subclavian vein [7]. The vein then continues below the clavicle going towards the sternal notch until it reaches the medial border of the anterior scalene muscle [7]. Therein, the subclavian vein joins the internal jugular vein and becomes the brachiocephalic vein.

**Figure 3:**
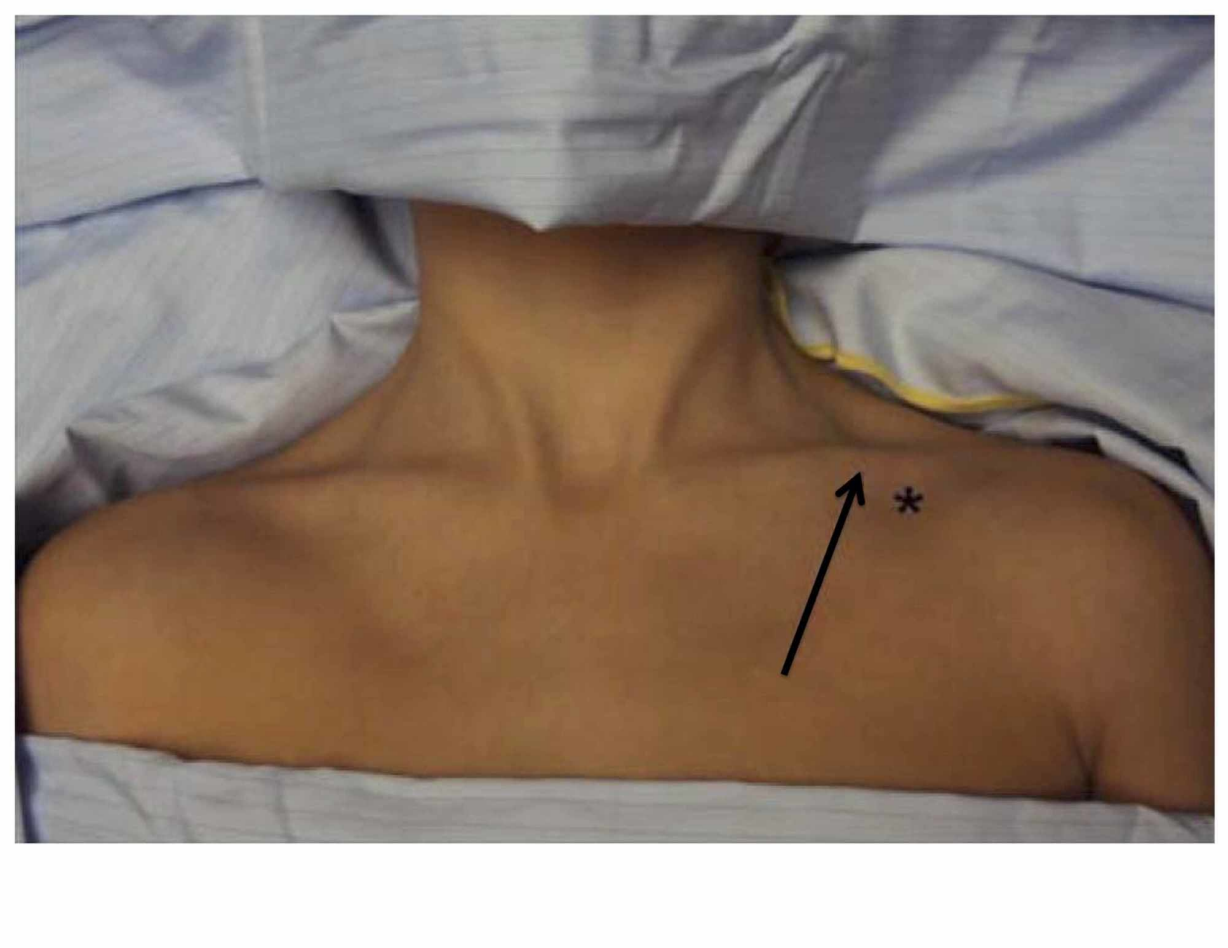
The site of a central line insertion into the subclavian vein The needle for the venous catheter must pass immediately beneath the junction of the medial one-third and lateral two-thirds of the clavicle (pointed out by the black arrow). The puncture must occur at a point 1-2 cm inferior and lateral to the junction (as marked by the black star) [3].

A central line insertion is usually done by the infraclavicular approach where the physician stands on the same side as the desired vein and turns the head of the patient to face the opposing direction [8]. The skin is then punctured one centimeter caudal to the junction of the medial and middle one third of the clavicle with the needle pointed towards the sternal notch (Figure 3) [8]. Right-sided subclavian central lines malfunction less often than left-sided subclavian central lines [9].

The Internal Jugular Vein

The internal jugular vein is another site of central line placement. The internal jugular vein arises from the sigmoid sinus in the posterior cranial fossa and then exits the cranium through the jugular foramen [10-12]. Then it descends further and at the junction of the neck and the thorax, it will join with the subclavian vein to form the brachiocephalic vein. Several important structures are located near the vein during its anatomical course. During its course the internal jugular vein is anterior to the vagus nerve. It also lies just lateral and anterior to the internal and common carotid arteries [10-12]. Any misstep in a central line insertion would increase the risk of injury to these structures. 

**Figure 4:**
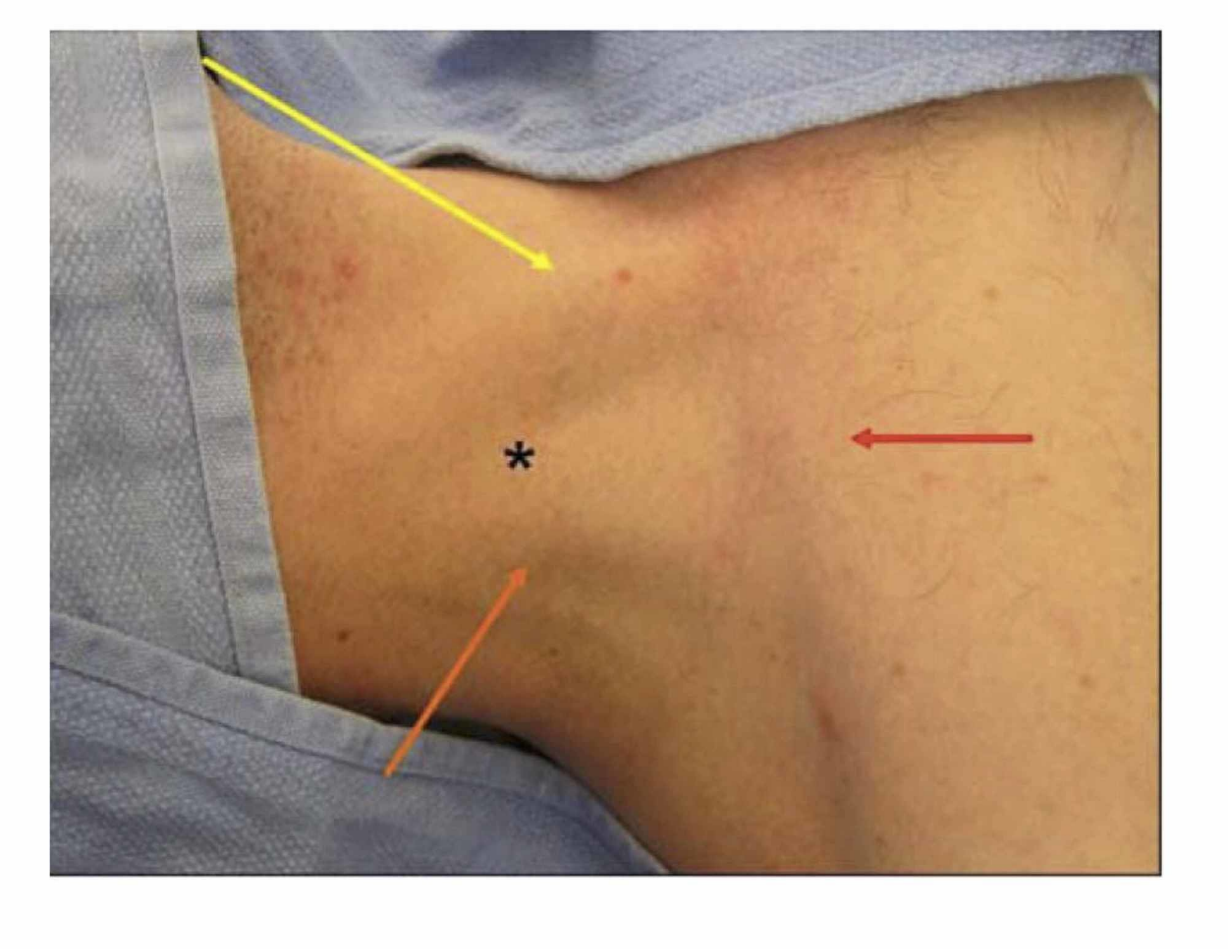
The site of a central line insertion into the internal jugular vein For the site of a central line insertion, the essential surface anatomy comprises the borders of Sedillot’s triangle. Sedillot's triangle comprises the sternal head of the sternocleidomastoid muscle medially (pointed out by the yellow arrow), the clavicular head of the sternocleidomastoid laterally (pointed out by the orange arrow), and the superior border of the medial third of the clavicle inferiorly (pointed out by the red arrow) [3]. The position of the appropriate cutaneous puncture is at the apex of the triangle (as marked by the black star).

The insertion of a central line in the internal jugular vein was previously done by the central approach where a needle is pointed to the ipsilateral nipple and a puncture is done at the apex of the triangle (Figure 4) [8]. With the increased use of ultrasound guided insertion, the central approach technique has been made obsolete. The right jugular vein is more commonly used than the left because the right jugular vein drains immediately into the superior vena cava and the left does not. Also the apex of the left lung is located at a higher level than the right lung. These anatomical differences mean that the left jugular central line has a higher risk of developing pneumothorax [8].

The Femoral Vein

The common femoral vein, otherwise known as the femoral vein, is the last of the most common sites of central line insertion. The femoral vein receives drainage from the joining of the superficial femoral vein and the deep femoral vein in the upper thigh. Once it reaches above the inguinal ligament, the femoral vein continues as the external iliac vein. The internal iliac vein combines with the external iliac vein to form the common iliac vein which eventually combines with its opposite side counterpart to become the inferior vena cava (IVC). The IVC drains into the right atrium [13].

The femoral vein is located in the femoral triangle. This is an anatomical region bounded by the inguinal ligament superiorly, the adductor longus medially, and the sartorius muscle laterally. The physician will first palpate the femoral artery by using the inguinal ligament and an anatomical point midway between the anterior superior iliac spine and the pubic tubercle. Once the pulsation is felt, the location is easily determined because the femoral vein is always medial to the femoral artery within the femoral triangle [13]. If the findings need to be confirmed before proceeding then an ultrasound can be used to guide the procedure. 

The complications associated with a central line insertion

There are several complications associated with central line use, regardless of the site of insertion. Past research has demonstrated that ultrasound guidance has been shown to decrease the risk of complications at all central line access sites [2,9].

The Immediate Complications

Complications that occur during or closely following a central line insertion are called immediate complications. The complications are categorized into the following; cardiac, vascular, pulmonary, and catheter placement complications [14]. These are caused by errors made during a central line insertion procedure [14]. So in order to reduce the occurrence of these complications, it becomes important to address these errors, how they cause the complications, and how they are managed. In 1986, a group of physicians conducted a prospective study and recorded 714 attempts at central venous catheterization during an eight-month period of time in the intensive care unit (ICU) [15]. The rates of catheterization failure and the early complications (amongst the three common sites) were determined. The procedures were conducted by two groups, the first being staff members and experienced residents, and the second being interns and inexperienced residents. The overall failure rate was 10.1% for the experienced group and 19.4% for the inexperienced group. The complication rate was 5.4% for the experienced group and 11% for the inexperienced group [15]. As mentioned earlier, reducing the rate of immediate complications means reducing the errors made during the procedure. One such improvement has been the use of ultrasound-guided central line insertion and its effect on reducing the immediate complication rates. In a 2011 study, it was determined that ultrasound guidance during insertion significantly reduced the incidence of immediate complications from rates previously as high as 11.8% which then reduced to four percent and later became seven percent [16]. 

Cardiac complications: Cardiac complications are considered to be one of the immediate complications seen in a central line insertion. Physicians will encounter arrhythmias during or as an immediate result of the insertion because of the guide wire coming into contact with the right atrium [14]. As a result, premature atrial and ventricular contractions can occur [14]. Having awareness of the guide wire depth and using telemetry monitoring will help in the early recognition of arrhythmia. If it does develop, then advanced cardiac life support (ACLS) should be started immediately [14]. If the atrioventricular (AV) node is contracting for a significant amount of time then supraventricular tachycardia can occur and that can lead to a fatal arrhythmia and cardiac arrest [15,17-18]. Other cardiac complications include right ventricular perforation caused by inserting a pulmonary artery catheter, which will lead to a cardiac tamponade [14]. This will require a fast assessment and prompt removal of the patient to the operating room for a pericardiocentesis.

Vascular complications: The vascular complications seen during a central line insertion are arterial injury, venous injury, bleeding, and hematoma formation [14]. Ultrasound guidance has been shown to greatly reduce the risk of vascular complications [17,19-21]. Arterial injury tends to occur most commonly in cases of femoral vein central lines and least commonly in subclavian vein central lines [22].

It was determined in a 2014 study that arterial punctures occur in about 4.2% to 9.3% of all central line placements [23-24]. It is often recognized secondary to its characteristic pulsatile flow but it may be difficult to diagnose in patients who have a low blood volume [23-24]. And despite the use of ultrasound, there are still central lines that end up in the arterial system by accident [25]. The concern in that case is whether to immediately remove the catheter with pressure or leave it in place [14]. Both carry risk. Any prolonged arterial catheterization can lead to a stroke, a thrombus, and potential neurological problems. The immediate removal of an accidental arterial catheter with pressure can lead to the possible development of hemorrhage, a pseudoaneurysm, or an AV fistula [14]. The risk of hemorrhage is even greater in patients on anticoagulants or antiplatelet drugs [23]. Past research studies have determined that leaving the accidental arterial catheter in place with a quick repair will carry less morbidity and mortality rather than performing catheter removal with pressure [26]. Pseudoaneurysms are treated with compression or image-guided thrombin or coil placement. An AV fistula would be managed with an image-guided coiling procedure [14]. But if the treatment is delayed for a significant amount of time, then a direct surgical repair may be needed as a last resort.

Venous injuries can also occur during a central line insertion. Past studies have reported lacerations of the vena cava, the mediastinal vessels, and the right atrium [14]. It has been hypothesized that the during the insertion, the guide wire becomes trapped against the vessel wall and the subsequent insertion of the dilator or catheter leads to that wire bending and pushing against the vessel wall [14]. This can lead to a laceration injury. A direct visualization of the guide wire using fluoroscopy can help prevent atria or venous injury [23]. Sometimes for the treatment of these injuries, surgery is performed, which can range from a direct suture repair to a complete vascular reconstruction using either autologous tissue or bovine pericardium tissue [14]. The difficulty with using prosthetic material in the venous system is that it is highly thrombogenic even if the patient is taking anticoagulant medication [14]. In the case of a venous injury resulting in life-threatening hemorrhage, urgent ligation of the bleeding vessel is done immediately.

Hematoma formation has also been reported in about 4.7% of all central line insertions [24]. Most hematomas formed during central line insertions are benign but some can become sources of infection in patients and lead to abscess formation. Blood can collect in the thorax or in the mediastinum leading to hemothorax or hemomediastinum, respectively. They would require treatment with computed tomography (CT) drainage. 

Abnormal anatomy in patients can also predispose to vascular complications occurring during a central line insertion. In about 0.3% of healthy patients, there is a congenital persistence of a left-sided vena cava, with or without a bridging innominate vein [27-28]. The significance of a left-sided vena cava is that it will drain directly into the left atrium versus into the coronary sinus [28]. The potential complications of a central line insertion in this situation are the development of systemic emboli and cardiac arrhythmias [28]. The anomaly can be incidentally discovered during a central line insertion in the left subclavian vein or the left internal jugular vein [27-28]. When it is recognized, the central line should be quickly removed and placed in either the right subclavian vein or internal jugular vein. 

Device mismanagement during insertion: There have been reported cases of catheter and wire entanglement with IVC filters. In most cases, fluoroscopic visualization is utilized to correct the IVC filter entanglement [29-30]. Other reported cases have seen catheter entanglement in patients with multiple catheters or by entrapment with sutures during cardiothoracic surgery [31]. The entanglement often results in what physicians refer to as knotted catheters. In order to remove them, physicians will tighten the knot (of entanglement) and remove the knot gently through a dilated insertion site. Another minimally invasive technique would be to manipulate the knot under fluoroscopy. In certain cases, surgical intervention may be resorted to [32-33]. Guide wires during insertion can also become entrapped or even lost. [34-36]. Entrapped or lost wires can often be resolved with surgical intervention, traction removal, or with the use of fluoroscopic guidance [35-36].

Pulmonary complications: The development of a pneumothorax, a pneumomediastinum, a chylothorax, a tracheal injury, a recurrent laryngeal nerve injury, and an air embolus are among the pulmonary complications seen during a central line insertion [14]. Any injury to the parietal pleura during insertion will lead to pneumothorax or pneumomediastinum formation. They are seen most commonly with subclavian vein central lines and occur in about one percent of cases [16,22,24]. A larger sized catheter and an increased number of attempted insertions will raise the risk for pneumothorax.

Other pulmonary complications seen are chylothorax and chylopericardium [37]. These conditions can be caused by venous congestion or an injury to the lymphatics. The left internal jugular vein and subclavian vein have a higher risk of lymphatic injury due to the anatomic location of the thoracic duct [14]. In the superior mediastinum, the thoracic duct passes behind the left internal jugular vein. The course of the thoracic duct finally terminates when it empties into the junction of the left subclavian and internal jugular veins. Despite this, a lymphatic injury can still occur with a central line inserted into the right internal jugular vein or subclavian vein. Lymphatic injuries can be treated with nitric oxide, thoracoscopic fibrin glue, or percutaneous coiling [17]. 

Recurrent laryngeal nerve injuries have had an incidence of 1.6% with central line insertions [38]. It is due to accidental trauma or perineural hematoma formation [14]. Other nerves that have also been injured are the sympathetic chain, the brachial plexus, and the phrenic nerves. The recovery time for an injury to the recurrent laryngeal nerve can take between six months and one year [14, 17, 38]. 

Past research studies have also noted the incidence of tracheal injuries during a central line insertion. They were usually due to an accidental puncture of the trachea while trying to gain vascular access with either a “finder” needle or a larger bore needle used to advance the guide wire [14, 39]. Surgical repair is needed for treatment [14].

The last known pulmonary complication is the development of an air embolism. These can occur during a central line insertion or when flushing the venous catheter. Small air embolisms (less than several cubic centimeters) are of little significance and can be self-resolving. If treatment is issued for them, then supplemental oxygen and increased systemic pressures are utilized to reduce the air bubble [40]. In extreme cases, hyperbaric oxygen therapy may be used to reabsorb the air [41]. 

The Delayed Complications

The delayed complications of a central line insertion include infection and device dysfunction. These complications are much more gradual in onset and can occur in the weeks to months after a central line insertion [14].

Infections: Infections of the central line can lead to sepsis, shock, and death. The incidence of a central line-associated infection is between 80-189 episodes per 100,000 patient years [42]. The additional average cost of each infection is about $16,550 [42]. The reported patient mortality rate was between 12% and 25% [42]. Infections are linked to biofilm formation on the venous catheter with *Staphylococcus aureus* and *Staphylococcus epidermidis* bacteria being the two most common pathogens [14,43]. If a central line bloodstream infection is suspected, then two blood cultures should be drawn from separate sites before starting broad-spectrum antibiotics [14]. Broad-spectrum antibiotics should be given according to culture sensitivity [44].

Device dysfunction: A device dysfunction is when there is a problem with the mechanical components of the central line. A dysfunction of one them can lead to delayed complications like a fibrin sheath, a catheter fracture, a thrombosis, stenosis, or an infection. The rate of device dysfunction is directly related to the central line site, the duration, and the underlying patient comorbidities. The development of a fibrin sheath can occur within the first week of central line insertion and can create blockages at the distal openings. This will reduce the ability to draw blood from the line. For treatment, fibrinolytics such as alteplase can be prescribed to dissolve the fibrin sheath and where fibrinolytics fail, line stripping can be attempted [16,38]. 

A central line venous catheter fracture is another dysfunction seen most commonly with subclavian lines after a catheter has been in place for an extended period of time [14]. A catheter fracture can lead to life-threatening conditions like sepsis, endocarditis, cardiac perforation, or arrhythmia development [39]. The fracture is a consequence of pinch-off syndrome [14,17]. Pinch-off syndrome develops from the catheter being compressed by the subclavius-costoclavicular complex formed between the clavicle and first rib [14,17]. This compression will lead to the malfunction of the catheter thus increasing the risk of a catheter fracture [17]. Prompt and careful removal of all catheter parts is essential to prevent further damage.

Extended period central lines also have an elevated risk of causing venous thrombosis. Patients will have symptoms of ipsilateral extremity erythema, edema, and paresthesia [14]. The thrombosis can also lead to superior vena cava syndrome (SVC) in patients. The incidence of SVC syndrome in patients is one in every 1,000 cases [17]. Subclavian central lines have the lowest rate of thrombosis and femoral vein central lines have the highest rate of thrombosis [17,22]. Furthermore, cancer patients have amongst the highest risk of thrombosis at 41% [17]. 

A long-standing central line can lead to the development of venous stenosis. The risk of venous stenosis has a prevalence of 41% [38]. It is usually asymptomatic, but if symptomatic then the patients can be treated with stenting [38]. 

Peripherally inserted central catheters complications: In past research studies, it was hypothesized that peripherally inserted central catheters (PICC) had lower rates of infection as compared to central lines however current studies have not demonstrated a difference [45]. Despite the lack of clinical trial data comparing PICC lines with central lines, similar complications are seen in both. Past research studies have reported venous catheter fracture, embolization, cardiac perforation, cardiac tamponade, arrhythmias, and pneumothorax [46-50]. A PICC line insertion can also cause vascular complications like AV fistula formation and venous injury [14].

## Conclusions

The material reviewed in this paper focuses on central lines, their different types, the sites of insertion, and the complications seen in association with them. It goes into detail regarding the complications of central lines and how they are caused and treated. By understanding these parameters better, physicians can improve the way they handle central line insertions, especially if things go wrong. Despite this knowledge, larger studies are needed to better understand the complications of central lines and their compared efficacy to PICC lines. This is a review article for busy physicians to have a cumulative view of our current situation regarding the complications of central lines and where we are on our ability to effectively counteract them in the clinical setting.

## References

[1] Bell T, O'Grady NP (2017). Prevention of central line-associated bloodstream infections. Infect Dis Clin North Am.

[2] Bannon MP, Heller SF, Rivera M (2011). Anatomic considerations for central venous cannulation. Risk Manag Healthc Policy.

[3] Akaraborworn O (2017). A review in emergency central venous catheterization. Chin J Traumatol.

[4] Claure-Del Granado R, Mehta RL (2016). Fluid overload in the ICU: evaluation and management. BMC Nephrol.

[5] Hanafusa N (2015). Application of continuous renal replacement therapy: what should we consider based on existing evidence?. Blood Purif.

[6] Parás-Bravo P, Paz-Zulueta M, Sarabia-Lavin R (2016). Complications of peripherally inserted central venous catheters: a retrospective cohort study. PLoS One.

[7] Deere M, Burns B (2019). Central Venous Access, Subclavian Vein. https://www.ncbi.nlm.nih.gov/books/NBK482224/.

[8] Taylor RW, Palagiri AV (2007). Central venous catheterization. Crit Care Med.

[9] Vascular Access 2006 Work Group (2006). Clinical practice guidelines for vascular access. Am J Kidney Dis.

[10] Mumtaz S, Singh M (2019). Surgical review of the anatomical variations of the internal jugular vein: an update for head and neck surgeons. Ann R Coll Surg Engl.

[11] Garner DH, Baker S (2019). Anatomy, Head and Neck, Carotid Sheath. https://www.ncbi.nlm.nih.gov/pubmed/30137861/.

[12] Dublin AB, Al-Dhahir MA (2019). Anatomy, Head and Neck, Temporal Region. https://www.ncbi.nlm.nih.gov/pubmed/29494103/.

[13] Castro D, Martin Lee LAM (2019). Femoral Vein Central Venous Access. https://www.ncbi.nlm.nih.gov/books/NBK459255/.

[14] Kornbau C, Lee KC, Hughes GD, Firstenberg MS (2015). Central line complications. Int J Crit Illn Inj Sci.

[15] Sznajder JI, Zveibil FR, Bitterman H, Weiner P, Bursztein S (1986). Central vein catheterization. Failure and complication rates by three percutaneous approaches. Arch Intern Med.

[16] Bhutta ST, Culp WC (2011). Evaluation and management of central venous access complications. Tech Vasc Interv Radiol.

[17] Kusminsky RE (2007). Complications of central venous catheterization. J Am Coll Surg.

[18] Centers for Disease Control and Prevention (2011). Vital signs: central line-associated blood stream infections--United States, 2001, 2008, and 2009. MMWR Morb Mortal Wkly Rep.

[19] Powell JT, Mink JT, Nomura JT (2014). Ultrasound-guidance can reduce adverse events during femoral central venous cannulation. J Emerg Med.

[20] Leung J, Duffy M, Finckh A (2006). Real-time ultrasonographically-guided internal jugular vein catheterization in the emergency department increases success rates and reduces complications: a randomized, prospective study. Ann Emerg Med.

[21] Fragou M, Gravvanis A, Dimitriou V (2011). Real-time ultrasound-guided subclavian vein cannulation versus the landmark method in critical care patients: a prospective randomized study. Crit Care Med.

[22] McGee DC, Gould MK (2003). Preventing complications of central venous catheterization. N Engl J Med.

[23] Bowdle A (2014). Vascular complications of central venous catheter placement: evidence-based methods for prevention and treatment. J Cardiothorac Vasc Anesth.

[24] Vats HS (2012). Complications of catheters: tunneled and nontunneled. Adv Chronic Kidney Dis.

[25] Pillai L, Zimmerman P, d’Audiffret A (2009). Inadvertent great vessel arterial catheterization during ultrasound-guided central venous line placement: a potentially fatal event. J Vasc Surg.

[26] Guilbert MC, Elkouri S, Bracco D (2008). Arterial trauma during central venous catheter insertion: case series, review and proposed algorithm. J Vasc Surg.

[27] Shah PM, Babu SC, Goyal A, Mateo RB, Madden RE (2004). Arterial misplacement of large-caliber cannulas during jugular vein catheterization: case for surgical management. J Am Coll Surg.

[28] Ratliff HL, Yousufuddin M, Lieving WR, Watson BE, Malas A, Rosencrance G, McCowan RJ (2006). Persistent left superior vena cava: case reports and clinical implications. Int J Cardiol.

[29] Schelling G, Briegel J, Eichinger K, Raum W, Forst H (1991). Pulmonary artery catheter placement and temporary cardiac pacing in a patient with a persistent left superior vena cava. Intensive Care Med.

[30] Duong MH, Jensen WA, Kirsch CM, Wehner JH, Kagawa FT (2001). An unusual complication during central venous catheter placement. J Clin Anesth.

[31] Andrews RT, Geschwind JF, Savader SJ, Venbrux AC (1998). Entrapment of J-tip guidewires by Venatech and stainless-steel Greenfield vena cava filters during central venous catheter placement: percutaneous management in four patients. Cardiovasc Intervent Radiol.

[32] Graff J, Gong R, Byron R, Hassett JM (1986). Knotting and entanglement of multiple central venous catheters. JPEN J Parenter Enteral Nutr.

[33] Bossert T, Gummert JF, Bittner HB, Barten M, Walther T, Falk V, Mohr FW (2006). Swan-Ganz catheter-induced severe complications in cardiac surgery: right ventricular perforation, knotting, and rupture of a pulmonary artery. J Card Surg.

[34] Wang HE, Sweeney TA (1999). Subclavian central venous catheterization complicated by guidewire looping and entrapment. J Emerg Med.

[35] Song Y, Messerlian AK, Matevosian R (2012). A potentially hazardous complication during central venous catheterization: lost guidewire retained in the patient. J Clin Anesth.

[36] Jalwal GK, Rajagopalan V, Bindra A, Goyal K, Rath GP, Kumar A, Gamanagatti S (2014). Percutaneous retrieval of malpositioned, kinked and unraveled guide wire under fluoroscopic guidance during central venous cannulation. J Anaesthesiol Clin Pharmacol.

[37] Teichgraber UK, Nibbe L, Gebauer B, Wagner HJ (2003). Inadvertent puncture of the thoracic duct during attempted central venous catheter placement. Cardiovasc Intervent Radiol.

[38] Khouzam RN, Soufi MK, Weatherly M (2013). Heparin infusion through a central line misplaced in the carotid artery leading to hemorrhagic stroke. J Emerg Med.

[39] Konichezky S, Saguib S, Soroker D (1983). Tracheal puncture. A complication of percutaneous internal jugular vein cannulation. Anaesthesia.

[40] Hsu M, Trerotola SO (2015). Air embolism during insertion and replacement of tunneled dialysis catheters: a retrospective investigation of the effect of aerostatic sheaths and over-the-wire exchange. J Vasc Interv Radiol.

[41] Huang YC, Huang JC, Chen SC, Chang JM, Chen HC (2013). Lethal cardiac arrhythmia during central venous catheterization in a uremic patient: a case report and review of the literature. Hemodial Int.

[42] Centers for Disease Control and Prevention (2011). Vital signs: central line-associated blood stream infections--United States, 2001, 2008, and 2009. MMWR Morb Mortal Wkly Rep.

[43] Early TF, Gregory RT, Wheeler JR, Snyder SO Jr, Gayle RG (1990). Increased infection rate in double-lumen versus single-lumen Hickman catheters in cancer patients. South Med J.

[44] Pronovost P, Needham D, Berenholtz S (2006). An intervention to decrease catheter-related bloodstream infections in the ICU. N Engl J Med.

[45] Al Raiy B, Fakih MG, Bryan-Nomides N (2010). Peripherally inserted central venous catheters in the acute care setting: A safe alternative to high-risk short-term central venous catheters. Am J Infect Control.

[46] Ioachimescu OC, Stoller JK (2006). An ectopic peripherally inserted central catheter ('ectoPICC'). Cleve Clin J Med.

[47] Orme RM, McSwiney MM, Chamberlain-Webber RF (2007). Fatal cardiac tamponade as a result of a peripherally inserted central venous catheter: a case report and review of the literature. Br J Anaesth.

[48] Burns KE, McLaren A (2009). Catheter-related right atrial thrombus and pulmonary embolism: a case report and systematic review of the literature. Can Respir J.

[49] Elsharkawy H, Lewis BS, Steiger E, Farag E (2009). Post placement positional atrial fibrillation and peripherally inserted central catheters. Minerva Anestesiol.

[50] David G, Gunnarsson CL, Waters HC, Horblyuk R, Kaplan HS (2013). Economic measurement of medical errors using a hospital claims database. Value Health.

